# Removal of metal ions from water using oxygen plasma

**DOI:** 10.1038/s41598-021-88466-3

**Published:** 2021-04-28

**Authors:** Sayma Khanom, Nobuya Hayashi

**Affiliations:** 1 Interdisciplinary Graduate School of Engineering Sciences, Department of Advanced Energy Engineering Science, Kyushu University, Fukuoka, Japan; 2 Department of Soil, Water and Environment, University of Dhaka, Dhaka, Bangladesh

**Keywords:** Environmental sciences, Engineering

## Abstract

Zinc ion dissolved in water is attempted to be removed by generating the oxides of zinc using the oxygen gas in dielectric barrier discharge (DBD) plasma system. The removal rate of zinc oxides’ production (ZnO and Zn(OH)_2_) are measured at different treatment periods by the oxygen plasma penetration in water. The removal rate of the deposit increases initially and then decreases with the treatment period. The maximum removal rate (29%) of zinc ion from water is achieved at the treatment period of 10 min, where pH is lower (7.4). From FTIR the generation properties of zinc oxide can be recognized. Initially the amount of the deposit increases with the ozone treatment period due to production of both ZnO and Zn(OH)_2_. After that, the production of Zn(OH)_4_^2-^ increases even when the total removal rate of the deposit decreases. Therefore, to remove zinc ion from water forming metal oxide deposit, the penetration amount of the active oxygens to the water must be controlled to keep the pH lower than around 7.5. Because with increasing pH amount of removal rate of zinc oxides’ deposit decreases. The pH of the zinc dissolved water treated by ozone depends on both zinc ion and ozone concentration in water.

## Introduction

Metal contamination is one of the most complex issues of today’s contamination problems. Excess amount of heavy metal ions dissolved in tap water induces many serious problems on the health of living bodies including human^1–4^. There are two main sources of heavy metals in water-natural and anthropogenic. Natural sources comprise volcanic activities, soil erosion, activities of living organisms, and weathering of rocks and minerals, whereas anthropogenic sources include landfills, fuel combustion, street run-offs, sewage, agricultural activities, mining, and industrial pollutants, such as textile dyes^5^. In recent years, drastic increases in pollutants in water resources have been detected^6^. However, there is no effective and reasonable technique to remove heavy metal ions from the water.

Heavy metals are a group of trace elements that include metals and metalloids, such as arsenic, cadmium, chromium, cobalt, copper, iron, lead, manganese, mercury, nickel, tin, and zinc. They have a relatively high density of over 4 × 10^6^ mg/L. The metal ions are known to contaminate the soil, atmosphere, and water systems and are poisonous even in very low concentrations. Widespread uses of metals, the legacies of past contamination, and new technologies continue to pose important ecological risks for aquatic environments across the earth^7^. Especially, metal ions taken up by plants accumulate on each part of plants, and cause health problems on animals those ingest the plants^8–11^. Several methods have been used to remove heavy metals from contaminated water. They include chemical precipitation, ion exchange, adsorption, membrane filtration, reverse osmosis, solvent extraction, and electrochemical treatment^12–17^. Many of these methods suffer from high capital and operational costs. The rapid growth in world population brings with it the need for improvement in the current technology for water purification, in order to provide adequate potable water to everyone.

Advanced oxidation processes (AOPs) exhibit advantages over conventional treatment methods because of the generation of strong oxidants^18–20^. Among the AOPs, electrical discharge plasma is widely employed for wastewater treatment. Non-thermal plasma or low-temperature plasma is green technology and has been studied for water treatment^21–24^. Most studies have focused on using low-temperature plasmas to be inactivated microorganisms and to decompose organic compounds for wastewater treatment^25–27^. For water treatment, dielectric barrier discharge (DBD) have been commonly generated with the coaxial electrode configuration. When the DBD occurs using atmospheric air, energetic free electrons, ultraviolet (UV) light and variety of active species are produced in the electrode gap^25^. These species have short lifetime unlike other chemical reagents for water treatment.

Oxidation of the heavy metal ions using oxidation reagents sometimes produces deposits of metal oxide^28–30^. These metal oxide deposits can be removed from the water easily using filtration or deposition^31,32^. Active oxygen species such as oxygen radicals, OH radical and ozone, which have been used for AOPs, would also oxidize metal ions in liquid effectively. Among these species, ozone is one of the relatively stable active species. The ozone treatment of the tap water, which has been developed and put to practical use, would be also effective for the removal of metal ions from the water. This method is considered to be a new experiment where attempts are being made to eliminate metal ions from water using oxygen in DBD plasma method. However, low-temperature plasmas using oxygen, or any other gas has not yet been tested to remove metal ions from water treatment. In the present study, removal of metal ion from tap water is attempted by the ozone oxidation method using the DBD plasma. In this experiment, the zinc ion in water, as a typical example of metal ions dissolved in water, is attempted to be removed generating the zinc oxide using the ozone oxidation treatment.

## Experimental methods

**Figure 1:**
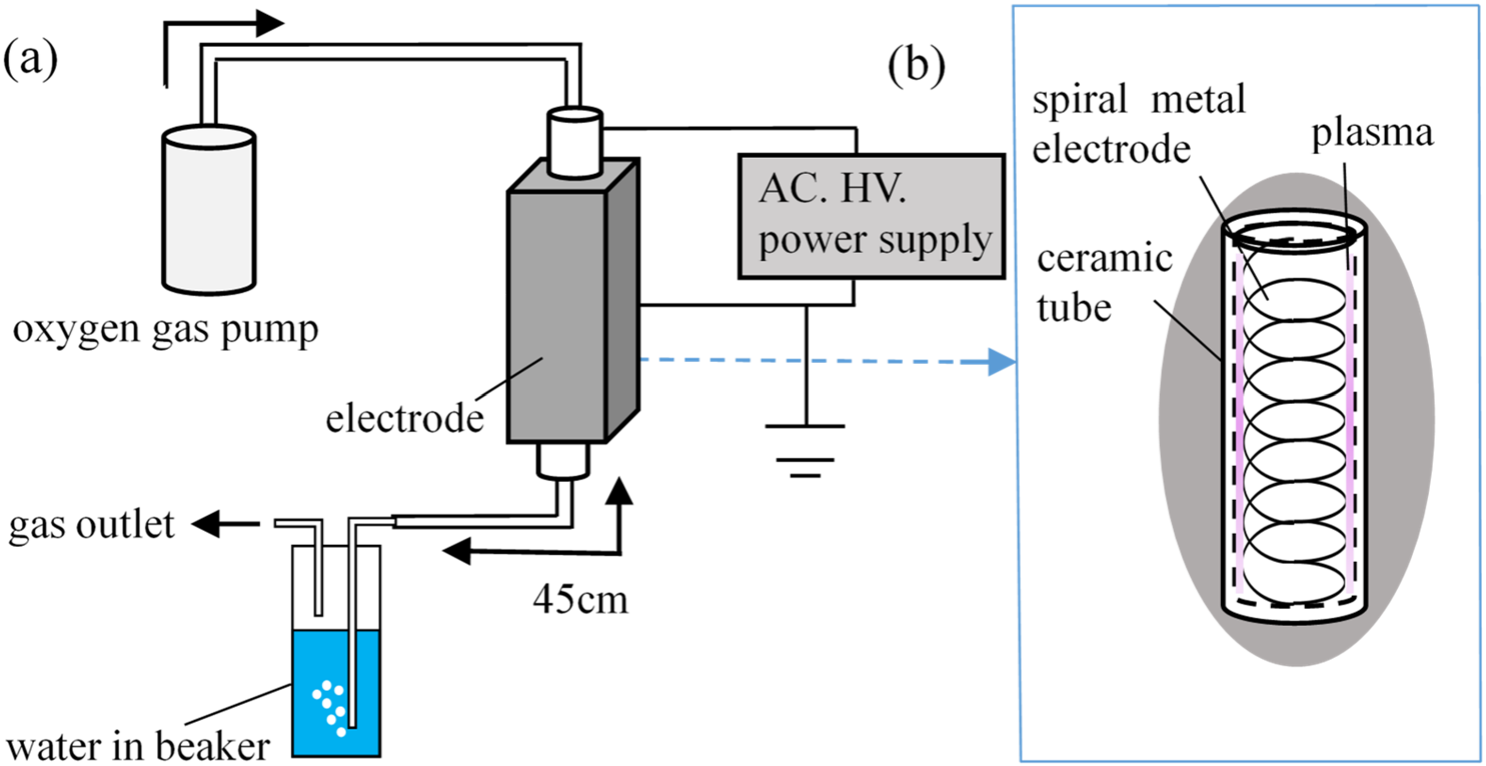
(**a**) Schematic diagram of the experimental apparatus, and (**b**) discharge electrode inside tube.

Zinc ion in water is produced by the electrolysis using cylindrical electrodes with 5 mm in diameter and 30 mm in length, which are made of pure zinc. Amount of water used in this experiment is 0.2 L. The DC voltage at 8.0 V is applied to one electrode, and then the zinc ion dissolves in water. The zinc concentration was measured by the colorimetric method using a color-developing reagent for zinc ion measurement. The color variation of the color-developing reagent is quantified using a photonic multichannel spectrometer and is converted into the zinc concentration using the standard curve of liquid color and zinc concentration. The ozone oxidation of the dissolved zinc ion is performed by the simple ozone bubbling in the water containing the zinc ion, as shown in Fig. 1a. The amount of water is 0.2 L and there is no water flow in the water vessel. The ozone is generated by the torch-shaped dielectric barrier discharge (DBD) using the pure oxygen gas with the flow rate of 1.0 L/min. The barrier discharge plasma torch used in this experiment has the shape of a cylindrical tube with dimensions of 100 mm in length and 4 mm in inner diameter, which is made from porous alumina. The cylindrical spiral-type discharge electrode is set along the inner wall of the alumina tube, and the copper film as grounded electrode is wounded on the outer of the alumina tube, as shown in Fig. 1b. When the high voltage with 5.0 kHz is applied to the discharge electrode, the barrier discharge occurs on the inner surface of the tube, and the oxygen plasma is generated.

**Figure 2:**
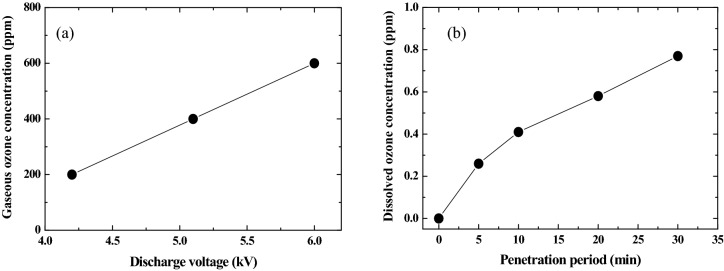
(**a**) Gaseous ozone concentration as a function of the discharge voltage of DBD, and (**b**) Dissolved ozone concentration at different penetration period of ozone in water.

The ozone is produced by high energy electrons in the plasma and is ejected from the opening edge of the tube by the oxygen gas flow. The produced ozone is transported into the water vessel and is dissolved in water using a bubbler. The concentration of the gaseous ozone ejected from the DBD device is measured using the gas detection tube and is controlled by changing the discharge voltage, as shown in Fig. 2a. This concentration range is almost same as that used for the practical ozone treatment of the tap water. Since the zinc ion dissolved in water interferences with the measurement reagent of the ozone titration, the ozone concentration dissolved in the pure water is measured as an index of the ozone concentration in the zinc ion water, as shown in Fig. 2b.

Effect of the oxidation of the zinc is evaluated by the concentration of the zinc oxide, which deposits on the bottom of the water. After the ozone treatment of the zinc dissolved water, the treated water in the conical tube is centrifuged, and then the supernatant of the water is removed. The precipitate with small amount of residual water is placed on the glass plate without washing and dried at 60 °C. Non-volatile materials dissolved in the water are included in the precipitate as well as the zinc oxide deposit. The weight of the dried deposit is measured using the precision measure and then calculate the removal rate from the deposit. In order to specify the composition of the deposit, the produced samples of precipitates are measured using the FTIR with the ATR mode, and the obtained spectra are compared with the typical spectra of zinc oxide.

## Results and discussion

### Discharge of zinc in water

Under normal conditions, zinc (Zn) does not react with water. In the high temperature circumstance of 600–800 °C, the zinc reacts with water to produce zinc oxide and hydrogen gas, as shown in the equation: Zn + 2H_2_O → ZnO + H_2_. The zinc oxide indicates yellow color at higher temperature, and white color at lower temperature.

In this study, the pure zinc electrode is used for the electrolysis of zinc ion in water. In a beaker a pair of zinc electrode is soaked in water of 0.2 L, and the DC voltage of 8 V is applied to an electrode for 60 min. The concentration of zinc ion in water is around 39 ppm. When electrical discharge occurs on the zinc electrode, the pH of the water increases with the discharge period according to following equations:

1 $${\text{Zn}} \to {\text{Zn}}^{2 + } + 2{\text{e}}^{ - }$$

2 $$2{\text{H}}_{2} {\text{O}} + 2{\text{e}}^{ - } \to {\text{H}}_{2} + 2{\text{OH}}^{ - }$$

Eq. (2) is hard to occur.

Reaction in Eq. (2) requires the higher pH condition. When the zinc discharge period increases, the pH of the water increases owing to the production of the OH^−^.

### Ozone reactions in water

When low-temperature plasma is formed using the DBD, high energetic free electrons, ultraviolet (UV) light and variety of active species are produced in the electrode gap^25^. Among these species, ozone is one of the most chemically stable and active species. The reactions to generate ozone in gas phase are expressed in Eqs. (3) and (4). In this study, the ozone generated in the gas phase is supplied in water for 120 min with the simple bubbling method. Dissolved ozone concentration in water is measured as a function of the penetration period of ozone in water, as shown in Fig. 2b. It is found that ozone concentration is increased with increasing treatment period.

3 $${\text{O}}_{2} + {\text{e}}^{ - } \to {\text{O}} + {\text{O}} + {\text{e}}^{ - }$$

4 $${\text{O}} + {\text{O}}_{2} \to {\text{O}}_{3}$$

In the water, ozone reacts with water molecule and the hydroxyl radical is produced as Eq. (5). The hydroxyl radical has very high reactivity and changes to hydroxide ion by receiving an electron from ions and molecules dissolved in water as impurities as Eq. (6). The hydroxide ion is stable in water.

5 $${\text{O}}_{3} + {\text{H}}_{2} {\text{O}} \to {\text{O}}_{2} + 2{\text{OH}}^{*}$$

6 $${\text{OH}}^{*} + {\text{e}}^{ - } \to {\text{OH}}^{ - }$$

The ozone dissociates to oxygen molecule and excited neutral atomic oxygen as Eq. (7). Also, the atomic oxygen changes to oxygen ion in water as Eq. (8).

7 $${\text{O}}_{3} \to {\text{O}}_{2} + {\text{O}}^{*}$$

8 $${\text{O}}^{*} + {\text{e}}^{ - } \to {\text{O}}^{ - }$$

These active oxygen species in the water would be the factor for the zinc oxides’ production (Zn(OH)_2_ and ZnO) from the zinc ion. The zinc oxide tends to coagulate and becomes deposit in water. Since the OH^*^ combines into the H_2_O_2_, the H_2_O_2_ concentration in the water is measured as the index of the OH^*^ generation. In this experiment no H_2_O_2_ is found in the zinc dissolved water.

### pH of water

**Figure 3:**
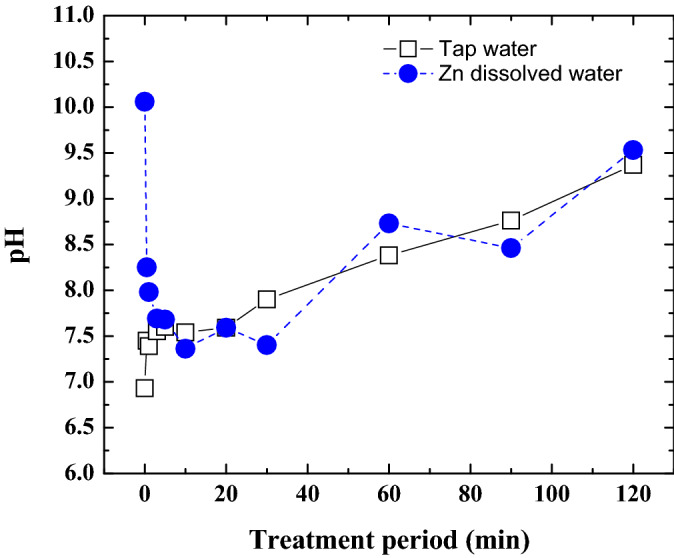
pH values of tap water and Zn dissolved water at different ozone treatment period.

The pH value of the water is one of the major factors for the deposition of metal oxides. Deposition of some metal oxides such as zinc and lead strongly depend on the pH value of the water. A significant role in the pH change can be attributed to positive and negative charges created in the DBD plasma that reach the water surface. The pH of tap water and Zn dissolved water are investigated changing the ozone penetration period till 120 min, as shown in Fig. 3.

The pH value of the ozone dissolved tap water till 10 min of the ozone penetration period increases rapidly owing to rapid increase of the ozone concentration. The pH increases monotonically with the ozone treatment period after 10 min of the ozone treatment period. When ozone dissolves in water, some portion of the ozone reacts with H_2_O, and hydroxyl ion (OH^−^) and oxygen (O_2_) are generated, as shown in Eq. (9). Electrons on the left side of the equation can be supplied from impurity in the tap water. Then, ozone dissolved tap water becomes alkaline condition.

9 $${\text{O}}_{3} + {\text{H}}_{2} {\text{O}} + 2{\text{e}}^{ - } \to {\text{O}}_{2} + 2{\text{OH}}^{ - }$$

In this experiment, when the oxygen plasma treatment period of the zinc dissolved water increases from 0 to 30 min, the pH of the zinc dissolved water changes from 10 to 7. Decrease of pH in Zn dissolved water occurs due to the Zn ion consumes the hydroxyl ions those are generated by the reaction of ozone with water. After 10 min, the pH of the Zn dissolved water increases with the ozone treatment period. The pH of the zinc dissolved water treated by ozone depends on both zinc ion and ozone concentration in water.

Opposite tendency of pH decreasing in water using oxygen plasma application were reported by different researchers^33–35^. Plasma acid–hydrogen cation H^+^ and superoxide anion O_2_^−^ is the cause of water acidification following plasma-treatment. In a direct system, the production of H^+^ ions can be a result of ion exchange mechanisms and the main conjugate base of the plasma treated water in oxygen is the superoxide radical. Thus, plasma acid may consist of hydrogen cation H^+^ and superoxide anion O_2_^–^ as the cause of water acidification following plasma-treatment^33–35^. In this study the changing in pH of water does not follow the previous studies. This may be due to attempts to remove metal oxide by indirect or remote plasma irradiation process.

### Ozone oxidation of zinc metal ion

**Figure 4:**
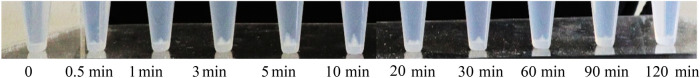
Deposits of zinc oxide compounds in water varying the ozone treatment period.

When the zinc ion dissolved in water contacts with ozone, white deposit of oxides of zinc are produced with the treatment period. Fig. 4 illustrates the photograph of the white deposit in a water, which is generated by the oxygen plasma penetration to the zinc ion dissolved water varying the ozone treatment period from 0 (without treatment) to 120 min. After the ozone treatment, the deposit is observed on the bottom of the tube. To investigate the tendencies of the production of zinc oxides in the water, the dried weight of the deposit obtained from the water is measured changing the oxygen plasma treatment period. Obtained deposit contains oxides of zinc, ZnO and Zn(OH)_2_. ZnO tends to deposit with lower pH, and Zn(OH)_2_ deposits with higher pH. With lower pH circumstance, zinc ion and oxygen ion produce the zinc oxide deposit as Eq. (10). When the pH increases, concentration of hydroxide ion increases. The zinc ion reacts with hydroxide ion and then zinc hydroxide is produced as Eq. (11).

10 $${\text{Zn}}^{ + } + {\text{O}}^{ - } \to {\text{ZnO}}$$

11 $${\text{Zn}}^{ + } + 2{\text{OH}}^{ - } \to {\text{Zn}}\left( {{\text{OH}}} \right)_{2}$$

**Figure 5:**
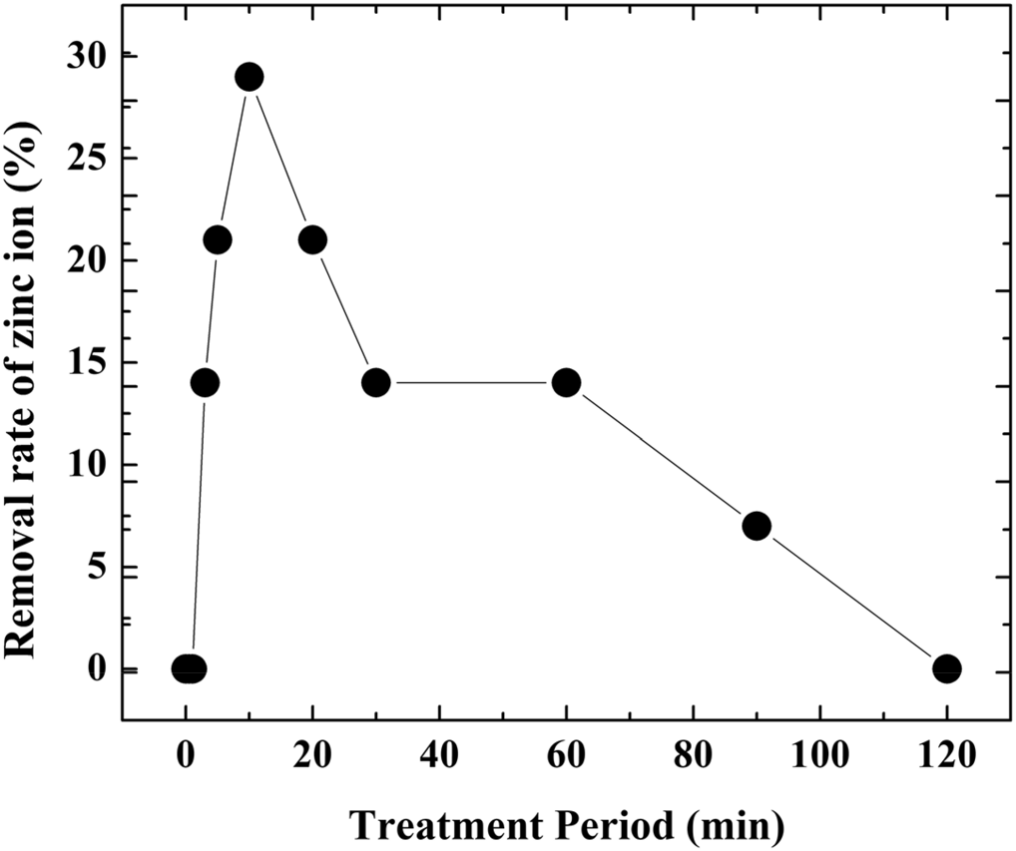
Removal rate of the zinc ion in the sample water changing the ozone treatment period.

Figure 5 indicates the removal rate of the zinc ion in the sample water changing the ozone treatment period. Removal rate of zinc ion increases with the treatment period almost linearly till 10 min. After 10 min the removal rate of zinc ion decreases with increasing the treatment period. The ozone treatment for 10 min shows maximum removal rate (29%) of the zinc ion, which is most suitable condition to produce the zinc oxide deposit. The dissolved ozone concentration is controlled by the discharge voltage of the DBD. The production of the zinc deposit increases with the dissolved ozone concentration till 0.41 ppm. When the treatment period increases till 10 min, reactions (12)–(14) occur. Since zinc ions consume the hydroxyl ions those are generated by the reaction of ozone with water in Eq. (9), pH of the water decreases. Then ZnO is dominant in the deposit.

12 $${\text{Zn}}^{2 + } + {\text{O}}^{*} + 2{\text{e}}^{ - } \to {\text{ZnO}}$$

13 $${\text{Zn}}^{2 + } + 2{\text{OH}}^{ - } \to {\text{Zn}}\left( {{\text{OH}}} \right)_{2}$$

14 $${\text{Zn}}\left( {{\text{OH}}} \right)_{2} \to {\text{ZnO}} + {\text{H}}_{2} {\text{O}}^{*}$$

When the treatment period becomes around 10 min, almost of the zinc ion changes to deposit, then OH^−^ starts to increase according to the Eq. (9). When the treatment period increases longer than 10 min and ozone is kept injected in the water even after removal of zinc ion from the water, OH^-^ increases and then pH of the water increases. The ZnO deposit becomes ionized and again dissolves in the water forming Zn(OH)_2_ and ${\text{Zn}}\left( {{\text{OH}}} \right)_{4}^{2 - }$. ${\text{Zn}}\left( {{\text{OH}}} \right)_{4}^{2 - }$ is the complex of Zn with OH^−^ ions, as shown in the Eqs. (15) and (16), under alkaline condition with pH higher than around 9. Therefore, to remove zinc ion from water forming metal oxide deposit, the penetration amount of the active oxygens to the water must be controlled to keep the pH lower than around 7.5. After 120 min of ozone penetration period, the zinc concentration is 10 ppm which is approximately 1/4th of the initial zinc concentration (39 ppm).

15 $${\text{Zn}}^{2 + } + 3{\text{OH}}^{ - } \to {\text{Zn}}\left( {{\text{OH}}} \right)_{3}^{ - }$$

16 $${\text{Zn}}^{2 + } + 4{\text{OH}}^{ - } \to {\text{Zn}}\left( {{\text{OH}}} \right)_{4}^{2 - }$$

In this experiment, the treatment is conducted under the practical condition. In ordinary case, tap water contains Ca and Mg ions with several ppm. In the tap water used in this experiment, the concentration of the Ca and Mg is typically 13.6 and 3.2 mg/l^36^. Ozone penetrated into the water is consumed partially by the oxidation of Ca and Mg. In this experiment, sufficient amount of ozone is penetrated into the water for the oxidation of Zn, Ca and Mg. Deposition condition of each metal oxide, which is produced by the ozone oxidation, is as follows: Mg(OH)_2_ can produce deposit with pH higher than 12, and Ca(OH)_2_ doesn’t generate deposit with any pH value. Also, Zn(OH)_2_ produces deposit with pH 9 to 11, and dissolves with pH higher than 12. In this experiment, the pH of the zinc dissolved water ranges from 7.4 to 10. Therefore, these facts indicate that deposit found in this experiment is totally Zn(OH)_2_, and then Ca and Mg don’t give influence on the removal rate of Zn.

**Figure 6:**
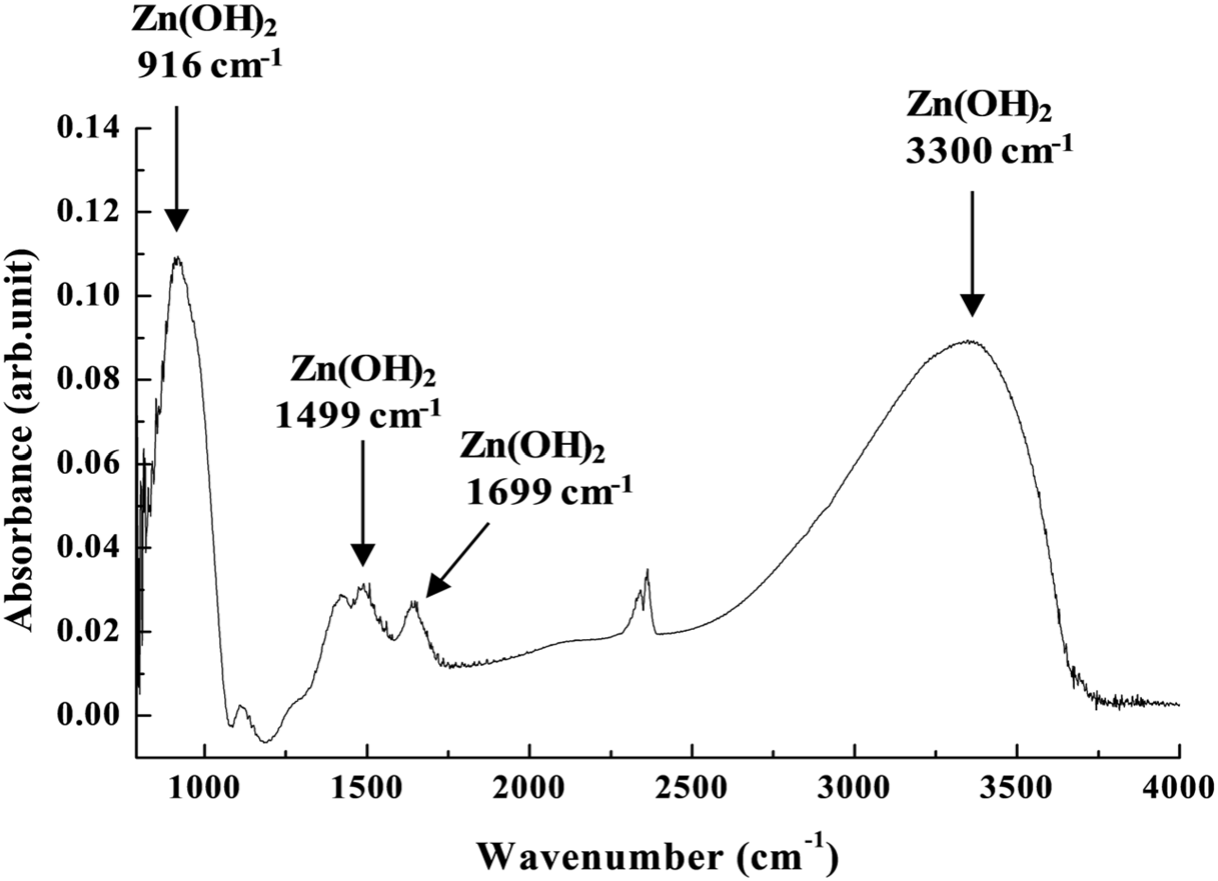
Typical IR absorbance spectrum of zinc oxide compounds deposit.

Figure 6 illustrates typical IR spectrum of the deposit extracted from the zinc ion dissolved water treated by the oxygen plasma penetration. Significant spectral peaks are assigned by the OH bonds in the zinc hydroxide. The zinc hydroxide in the deposit can be the Zn(OH)_2_. This result indicates that the ZnO, which is generated by the oxidation of zinc ion, has further been oxidized to the zinc hydroxides by the long-time penetration of the oxygen plasma. However, in this experiment, the zinc oxide peak is out of range of the FTIR spectrum. These obtained peaks are similar to those of the zinc hydroxide that is synthesized in water^34,35^. Therefore, the deposit, which is produced by the ozone oxidation of the zinc ion dissolved water, contains several oxides such as the ZnO, Zn(OH)_2_ and ${\text{Zn}}\left( {{\text{OH}}} \right)_{4}^{2 - }$. Also, from the obtained IR spectra, the significant peak of byproduct of the ozone treatment has not been observed on the spectrum.

**Figure 7:**
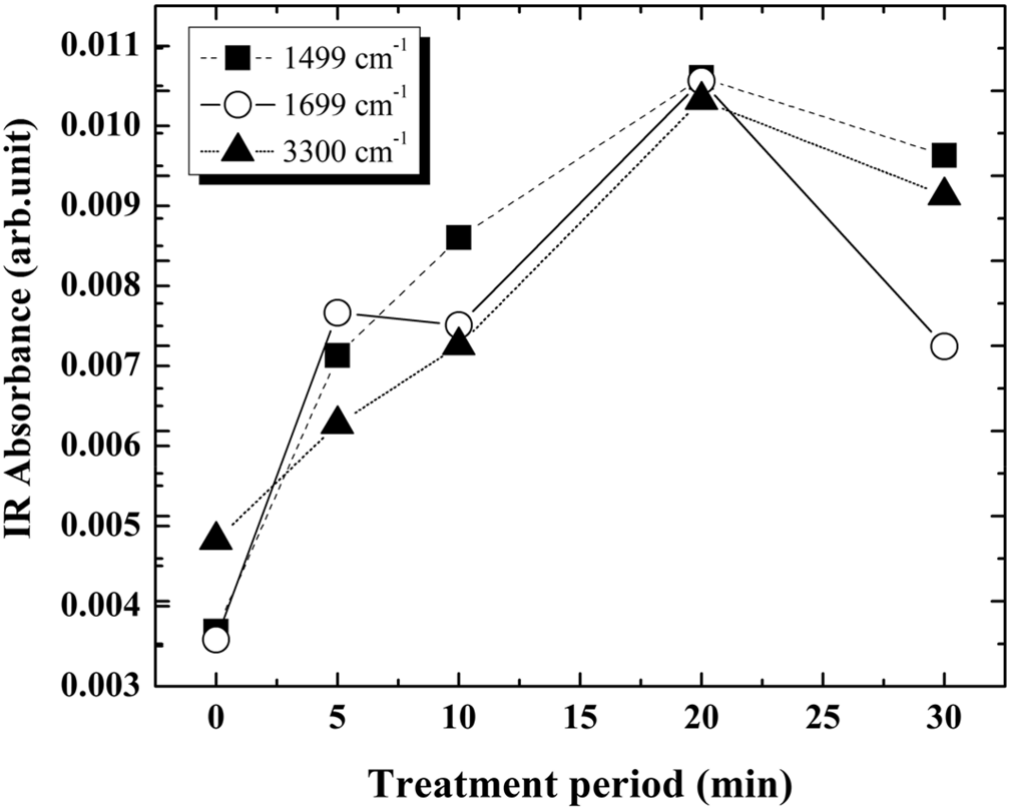
IR light absorbance by Zn(OH)_2_ contained in the deposit at different treatment period.

According to the Lambert–Beer’s law, the zinc oxide amount is proportional to the IR light absorbance of the zinc oxide. Figure 7 shows the IR light absorbance by the Zn(OH)_2_ contained in the deposit changing the treatment period, which is obtained from the peak height of the IR spectral peak of the deposit at around the wavenumber of 1499, 1699 and 3300 cm^−1^. The amount of the Zn(OH)_2_ in the deposit increases monotonically with the oxygen plasma treatment period up to 20 min and then decreases. Production characteristics of the zinc oxides’ can be clarified from the different tendencies of depositions shown in Figs. 5 and 7, which indicate generation properties of the ZnO + Zn(OH)_2_, Zn(OH)_2_ and ${\text{Zn}}\left( {{\text{OH}}} \right)_{4}^{2 - }$, respectively. The difference in the amount of deposits in these figures indicates the production tendency of zinc oxide. At 30 min the IR light absorbance decreases due to decrease of the Zn(OH)_2_ in the deposit. The amount of the ${\text{Zn}}\left( {{\text{OH}}} \right)_{4}^{2 - }$ increases constantly with the ozone penetration period, even when the total removal rate of the deposit decreases.

**Figure 8:**
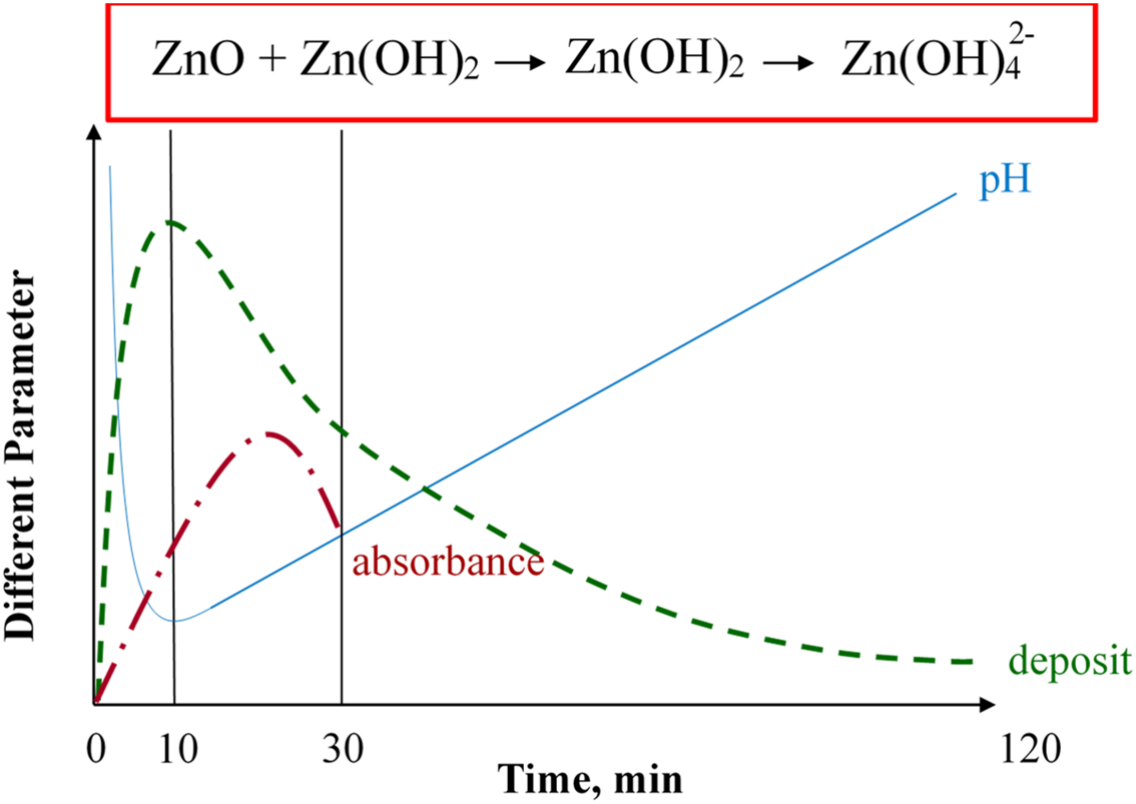
Schematic diagram of zinc oxide compounds with different parameter.

From this experiment, the maximum removal rate of zinc oxides’ compound deposits found at 10 min ozone penetration period where pH is lower, as shown in Fig. 8. When the treatment period becomes around 10 min, since zinc ions consume the OH^−^ those are generated by the reaction of ozone with water, pH of the water decreases. Then ZnO is dominant in the deposit. When the treatment period increases longer than 10 min, almost of the zinc ion changes into deposit, and then OH^−^ starts to increase and pH increases. The ZnO deposit becomes ionized and again dissolves in the water forming Zn(OH)_2_ and ${\text{Zn}}\left( {{\text{OH}}} \right)_{4}^{2 - }$. IR spectrum peaks also indicates that ZnO has further been oxidized to the zinc hydroxides by the long-term penetration of the oxygen plasma.

## Conclusions

Removal of zinc ion from water is attempted using the DBD oxygen plasma dissolved in water. Zinc ion in water can be removed from water as the zinc oxide deposit. The maximum removal rate (29%) of zinc ion from water is achieved at the treatment period of 10 min, where pH is lower (7.4). Therefore, to remove zinc ion from water forming metal oxide deposit, the penetration amount of the active oxygens to the water must be controlled to keep the pH lower than around 7.5. Because with increasing pH amount of deposit of zinc oxides’ decreases. The pH of the zinc dissolved water treated by ozone depends on both zinc ion and ozone concentration in water. Actually, precise control of the pH is not easy.
